# Heparanase overexpression impairs inflammatory response and macrophage-mediated clearance of amyloid-β in murine brain

**DOI:** 10.1007/s00401-012-0997-1

**Published:** 2012-06-13

**Authors:** Xiao Zhang, Bo Wang, Paul O’Callaghan, Elina Hjertström, Juan Jia, Feng Gong, Eyal Zcharia, Lars N. G. Nilsson, Lars Lannfelt, Israel Vlodavsky, Ulf Lindahl, Jin-Ping Li

**Affiliations:** 1 Department of Public Health and Caring Sciences, Molecular Geriatrics, Rudbeck Laboratory, University of Uppsala, Dag Hammarskjölds väg 20, 751 85 Uppsala, Sweden; 2 Department of Medical Biochemistry and Microbiology, The Biomedical Center, University of Uppsala, Box 582, Husargatan 3, 751 23 Uppsala, Sweden; 3 Department of Pharmacology, Institute of Pharmacology and Toxicology, Beijing, 100850 China; 4 Department of Blood Biochemistry and Molecular Biology, Institute of Transfusion Medicine, Beijing, 100850 China; 5 Cancer and Vascular Biology Research Center, The Rappaport Faculty of Medicine, Technion, 31096 Haifa, Israel; 6 Department of Pharmacology, Institute of Clinical Medicine, Faculty of Medicine, University of Oslo and Oslo University Hospital, Oslo, Norway

**Keywords:** Neuroinflammation, Heparan sulfate, Heparanase, Amyloid-β, Clearance, Alzheimer’s disease

## Abstract

Neuroinflammation is typically observed in neurodegenerative diseases such as Alzheimer’s disease, as well as after traumatic injury and pathogen infection. Resident immune cells, microglia and astrocytes, are activated and joined by blood-borne monocytes that traverse the blood–brain barrier and convert into activated macrophages. The activated cells express various cytokines, chemokines and proteolytic enzymes. To study the role of heparan sulfate proteoglycans in neuroinflammation, we employed a transgenic mouse overexpressing heparanase, an endoglucuronidase that specifically degrades heparan sulfate side chains. Neuroinflammation was induced by systemic challenge with lipopolysaccharide, or by localized cerebral microinjection of aggregated amyloid-β peptide, implicated in Alzheimer’s disease. Lipopolysaccharide-treated control mice showed massive activation of resident microglia as well as recruitment of monocyte-derived macrophages into the brain parenchyma. Microinjection of aggregated amyloid-β elicited a similar inflammatory response, albeit restricted to the injection site, which led to dispersion and clearance of the amyloid. In the heparanase-overexpressing mice, all aspects of immune cell recruitment and activation were significantly attenuated in both inflammation models, as was amyloid dispersion. Accordingly, an in vitro blood–brain barrier model constructed from heparanase-overexpressing cerebral vascular cells showed impaired transmigration of monocytes compared to a corresponding assembly of control cells. Our data indicate that intact heparan sulfate chains are required at multiple sites to mediate neuroinflammatory responses, and further point to heparanase as a modulator of this process, with potential implications for Alzheimer’s disease.

**Electronic supplementary material:**

The online version of this article (doi:10.1007/s00401-012-0997-1) contains supplementary material, which is available to authorized users.

## Introduction

Neuroinflammation is a prominent feature of several pathological conditions of the central nervous system (CNS), including neurodegenerative diseases such as Alzheimer’s disease (AD) [1, 25], CNS autoimmune diseases [5], traumatic brain injury [63] and pathogen infection [7]. Resident immune cells (microglia and astrocytes) are activated to release various cytokines, chemokines and proteolytic enzymes [38, 58]. Moreover, the cerebral parenchyma is invaded by blood-borne monocytes that traverse the blood–brain barrier (BBB) and convert into activated macrophages [16, 45]. The mechanisms behind these events are only partly understood, and the pathophysiological consequences of neuroinflammation are diverse and complex.

In AD, aggregated 40- or 42-residue peptides of amyloid-β (Aβ) deposit in the brain parenchyma as extracellular senile plaques and typically throughout the vasculature as cerebral amyloid angiopathy.

Seemingly contradictory effects of neuroinflammation have been noted in AD, on the one hand deposits appear to induce inflammatory reactions that recruit immune cells capable of dispersing the plaques, contributing to their clearance from the tissue [3, 52]. The relevance of these phenomena to the disease course is unclear, particularly so since soluble intermediate Aβ aggregates rather than fibrils have been directly implicated with neurotoxicity [20, 32]. Nevertheless, reactions leading to dispersal of Aβ deposits are potentially beneficial and merit characterization. Efficient Aβ phagocytes are believed to be derived from blood-borne monocytes that are recruited into the brain, where they differentiate to macrophages in the course of a complex neuroinflammatory response [9, 16]. The mechanism of cell recruitment across the blood–brain barrier (BBB) is only partly understood.

Previous studies demonstrated a key role for cell-surface heparan sulfate (HS) proteoglycans (HSPGs) in regulating leukocyte transmigration from blood to sites of inflammation [27, 33, 60]. The present study was undertaken to clarify the involvement of HS in neuroinflammation, induced either by systemic challenge with bacterial lipopolysaccharide (LPS) or by local deposition of Aβ fibrils in murine brain*.* We employed a transgenic mouse overexpressing heparanase (Hpa-tg), an endoglucuronidase that specifically degrades HS [11]. The Hpa-tg mice showed attenuated inflammatory response compared to control (Ctr) mice, and an impaired ability to disperse and clear Aβ aggregates. Moreover, HS and heparanase were directly implicated with regulation of monocyte transmigration across the BBB.

## Materials and methods

### Mice

The transgenic mouse overexpressing human heparanase (Hpa-tg), under the β-actin promoter, were generated according to Zcharia et al. [62], except that the enzyme was expressed as a chimer containing a chick heparanase signal peptide sequence, which promotes enzyme secretion [18]. The transgenic mice were backcrossed more than 10 generations on a C57BL/6 background and C57BL/6 was used as control (Ctr). The mice were used at 3–20 months of age. All experiments were approved by the regional animal research ethics committee.

### Determination of heparanase expression level

Heparanase ELISA was performed as described by Shafat et al*.* [50].

### Isolation and analysis of HSPG

Isolation and analysis of HSPG were performed as previously described [11]. See Supplementary methods for details.

### Lipopolysaccharide (LPS) treatment

Mice (4-month-old) were intraperitoneally injected with a single dose (5 mg/kg) of LPS (10 μg/100 μl PBS) (LPS L2280, derived from O55:B5 *E.coli*, Sigma-Aldrich). After 20 h the mice were deeply anesthetized, transcardially perfused with 50 ml saline, and the brains were dissected. One hemisphere of the brain was fixed in 4 % formaldehyde for cryotome tissue section preparation and the other was rapidly frozen in dry ice for preparation of tissue extract.

### Western blotting

Protein samples were separated by 10–20 % SDS-PAGE and then transferred to a nitrocellulose membrane. After blocking with 5 % nonfat dry milk, the membranes were probed with primary antibodies (see Supplementary Table 1 for an account of all antibodies used in the study) followed by the corresponding secondary antibodies. Signals were visualized using SuperSignal West Pico or Dura substrates (Thermo). Quantitative band analysis was performed with ImageJ software.

### Intracortical Aβ42 injection

The injection was performed on deeply anesthetized (2.5 % avertin 500 μl/mouse i.p.) mice under stereotaxic guidance with coordinates from the bregma: +2.0 mm anteroposterior, −2.0 mm lateral, and −2.3 mm dorsoventral. Five micrograms of aggregated synthetic human Aβ1-42 (PolyPeptide Laboratories GmbH, Germany) (5 μg/1 μl) was injected into one hemisphere at a rate of 0.2 μl/min, followed by a 2-min pause for absorption of the injected solutions. The brains were dissected as described above, fixed in 4 % formaldehyde overnight and processed according to standard protocols for the preparation of paraffin-embedded tissue blocks.

### Histochemistry and immunostaining

Immunostaining with antibodies against CD45, F4/80, CD31 and ICAM-1 were performed on 20 μm cryotome brain tissue sections. All other immunostainings and Congo red histochemistry were performed on 5 μm paraffin sections. After antigen retrieval, primary antibody incubation was carried out overnight at 4 °C followed by incubation with secondary antibody for 30–60 min at room temperature. ABC™ complex and NOVA™ red reagents (Vector Labs) were used to visualize the immunosignals. In another setting, a rat on mouse AP-polymer kit, developed with Vulcan Fast Red Chromogen kit2 (Biocare Medical) was employed. For double immunostaining, primary antibodies were incubated overnight, simultaneously or stepwise at 4 °C, followed by incubation with the appropriate Alexa fluor labeled secondary antibodies (Invitrogen). Hematoxylin and DAPI (4′,6-diamidino-2-phenylindole) were used for counterstaining of nuclei.

### Microscopy and image analysis

Microscopy was performed using a Nikon DXM1200F™ instrument (Nikon, Melville, USA). CD45-positive cells associated with microvessel walls or partially exited from vessels were counted under microscope and the number of such cells associated with 10 microvessels of similar length (~300 μm) in each section was summarized. Images of anti-F4/80 and anti-CD45 immunostained sections were captured and the positive cells were quantified with ImageJ software. The images were converted to 8-bit grayscale format and a standard segmentation threshold for positively stained pixels was established. Using the ImageJ particle analysis tool with a size setting of 8–20 pixels, the number of amoeboid and ramified CD45-positive cells was recorded and with a setting of 20–100 pixels, the number of F4/80-positive cells was recorded. Cross-sectional information and z-stacks for selected deposits were obtained by confocal laser scanning microscopy using a Carl Zeiss LSM 510 META™ instrument (Carls Zeiss, Germany).

### In vitro BBB model and monocyte transmigration

Primary endothelial cells and pericytes were prepared from the brains of Hpa-tg and Ctr mice. The in vitro BBB model was constructed on Falcon™ HTS FluoroBlok™ 3.0 μm colored PET Membrane Inserts for 24 well culture plates (BD). Pericytes (10,000 cells/insert) were grown on the basolateral side and endothelial cells (150,000 cells/insert) on the apical side of the membrane, which was pre-coated with bovine fibronectin. The cells were grown for 3 days prior to experiments. The integrity of the endothelial monolayer was confirmed by establishing that Evans blue-bound BSA did not diffuse from the apical to the basolateral compartment. GFP-monocytes (120,000 cells/insert) were, immediately after isolation, loaded into the apical compartment (blood), and 50 ng/ml of CCL2 was added to the basolateral compartment (brain). Monocytes migration from the apical to the basolateral compartment was monitored with a Zeiss laser scanning microscope LSM 700/CO2 Microscope with a stage incubator (37 °C, 5 % CO_2_). The number of GFP-monocytes was counted with ImageJ software. For details, see Supplementary methods.

### Statistical method

Two-tailed unpaired Student’s *t* test was used to determine the significance between population means. Statistical significance was set at *P* < 0.05.

## Results

### Cerebral expression of heparanase and altered HS structure in transgenic mice

**Fig. 1:**
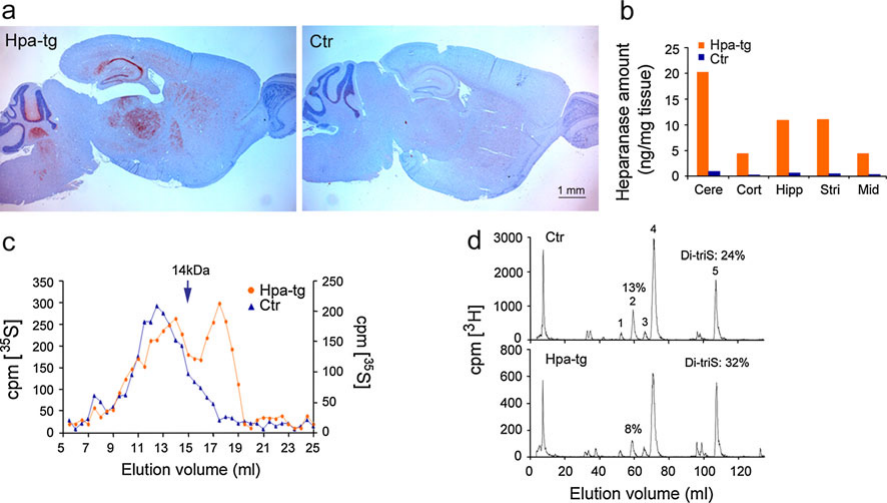
Changed structure of heparan sulfate in mouse brain due to heparanase overexpression. **a** Paraffin-embedded sagittal brain sections of Hpa-tg (*left*) and Ctr (*right*) mice were immunostained with anti-heparanase antibody 733 (*red*). **b** Heparanase levels in extracts of Hpa-tg and Ctr brain tissue, analyzed with ELISA. *Cere* cerebellum, *Cort* cortex, *Hipp* hippocampus, *Stri* striatum, *Mid* midbrain. **c** Gel chromatography (Superose 6) of metabolically ^35^S-labeled HSPG purified from Hpa-tg and Ctr brains. The elution position of heparin (14 kDa) is indicated by the *arrow*. **d** Anion-exchange HPLC (Partisil-10 SAX column) of disaccharides generated by deaminative cleavage of HS derived from brain tissue extracts of Hpa-tg and Ctr mice. The resulting disaccharides were radio-endlabeled by reduction with NaB_3_H_4_. *Numbered peaks* represent the following disaccharide units in the intact HS chains: *1* -GlcA2S-GlcNS-, *2* -GlcA-GlcNS6S-, *3* -IdoA-GlcNS6S-, *4* -IdoA2S-GlcNS-, *5* -IdoA2S-GlcNS6S-. The proportions of peaks *2* (the disaccharide cleaved by heparanase) and *5* (Di-triS: hypersulfated disaccharide) are indicated as percentage of total disaccharides

We have previously described a transgenic mouse overexpressing human heparanase, which results in extensive fragmentation of HS chains in vivo [11, 62]. To study the role of HS in neuroinflammation, we generated a new strain (Hpa-tg) that overexpresses a high level of heparanase in the brain. The transgenic enzyme was expressed, albeit to variable extent, in all examined regions of the brain (Fig. 1a, b). Gel chromatography of metabolically radiolabeled HSPG isolated from Hpa-tg brain detected HS fragments that were not present in Ctr brain tissue (Fig. 1c). Disaccharide compositional analysis revealed a decrease in the -GlcA-GlcNS6S- unit, representing the cleavable linkage target for heparanase (Fig. 1d; peak 2), and an increase in trisulfated -IdoA2S-GlcNS6S- disaccharide (Fig. 1d; peak 5), as previously ascribed to heparanase upregulation [11].

### Reduced cerebral inflammatory response to LPS challenge in Hpa-tg mice

**Fig. 2:**
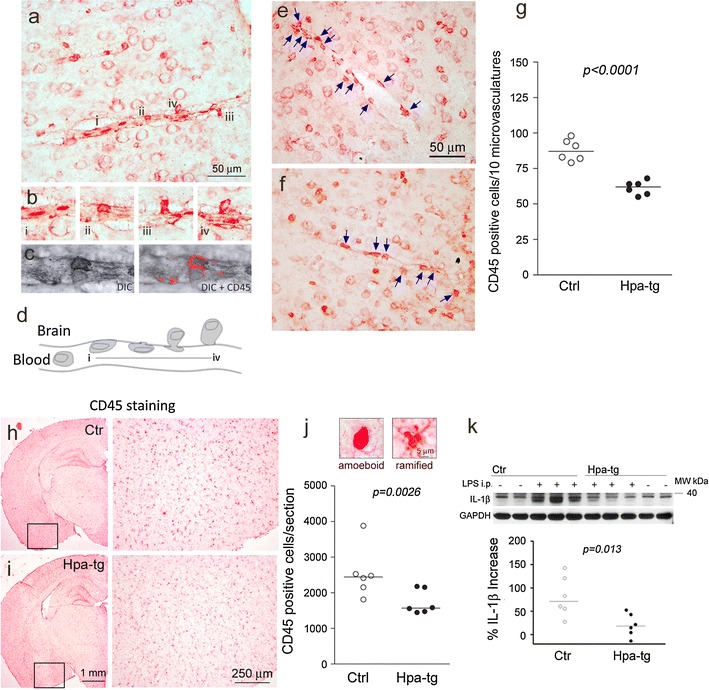
Impaired immune response of Hpa-tg brain to LPS challenge. Four-month-old Hpa-tg and Ctr mice received LPS (5 mg/kg) by intraperitoneal injection, and were killed 20 h later and brain tissue sections were immunostained with anti-CD45 antibody. **a**–**d** CD45-positive cells associated with microvascular structures in Ctr brain: **a** the morphologies of CD45-positive cells associated with the vasculature are consistent with those assumed by monocytes transmigrating from the blood to the brain parenchyma, converting to macrophages in the course of this process. **b** CD45-positive cells in (*i*) and (*ii*) remain intravascular but are associated with the vessel wall, whereas the cells in (*iii*) and (*iv*) have largely exited the vessel. **c** Confocal laser scanning microscopy of the CD45-positive cell in (*ii*) imaged with differential interference contrast (DIC). **d** Model of monocyte attachment and diapedesis from blood to brain, typically stimulated in response to CNS-derived chemokines. **e**–**f** CD45-positive cells (indicated by *arrows*) associated with Ctr and Hpa-tg microvasculature, respectively. **g** Significantly fewer CD45-positive cells are associated with Hpa-tg than with Ctr microvasculature. The data are presented, for each animal, as total number of CD45-positive cells from 10 microvasculatures, each of ~300 μm length. **h**, **i** Panels to the right show high-magnification images of the cortical areas indicated by *frames* in the *left panels*. **j** *Top* representative amoeboid (*left*) and ramified (*right*) CD45-positive macrophages from either Ctr or Hpa-tg sections; *bottom* Significantly fewer CD45-positive macrophages in Hpa-tg whole-brain tissue sections than in Ctr sections. **k** Western blotting of IL-1β in Hpa-tg and Ctr brain. Densitometry analysis of the bands demonstrated a significantly higher up-regulation of IL-1β in Ctr brains than in Hpa-tg brains following LPS challenge

LPS is a bacterial endotoxin known to upregulate cerebral expression of pro-inflammatory factors such as interleukin-1β (IL-1β) [22], tumor necrosis factor α (TNF-α) [44] and CCL2 (also designated monocyte chemotactic protein-1) [55]. Once released, the pro-inflammatory factors stimulate recruitment of neutrophils [47], and activation of microglia [44]. We examined the cellular response of the Hpa-tg brain to LPS treatment. Brain tissue sections of 4-month-old, LPS-challenged mice were immunostained with antibodies against CD45, a major protein tyrosine phosphatase located at the leukocyte plasma membrane [56] and F4/80, a glycoprotein cell-surface marker that is amply expressed by murine macrophages [2]. Anti-CD45 antibody strongly stained cells with amoeboid morphology in association with microvasculature (Fig. 2a, e–f) and in the parenchyma (Fig. 2j; left insert). This phenotype is primarily associated with macrophages derived from blood-borne monocytes [19], which was supported by the detection of CD45-positive cells at various stages of transmigration (Fig. 2b; i–iv), including cells attached to the luminal wall (Fig. 2c). The transmigration process is outlined in the graphic in Fig. 2d. To assess transmigration of blood-borne monocytes, we counted the CD45-positive cells associated with microvasculature (Fig. 2e, f) and found significantly more CD45-positive cells in Ctr than in Hpa-tg mice (Fig. 2g). Resident microglia are characterized by highly branched morphology and low levels of CD45 [41, 49] that are upregulated in the course of activation [14, 42]. We counted CD45-positive cells of ramified and amoeboid morphology (Fig. 2h–j; inserts) in whole tissue sections and found, again, significantly increased numbers of CD45-positive cells in Ctr compared to Hpa-tg mice (Fig. 2j). Immunostaining with anti-F4/80 antibody revealed a massive recruitment of activated macrophages in Ctr brains (Supplementary Fig. 1a: insert, b). Again, such cells were less abundant in Hpa-tg brains (Supplementary Fig. 1c). The abundant ramified macrophages presumably include activated resident microglia as well as cells derived from blood-borne monocytes. Mice of similar age, not challenged with LPS showed few CD45- and F4/80-positive cells, irrespective of Ctr or Hpa-tg status (Supplementary Fig. 1d-g). Consistent with these findings, protein levels of the pro-inflammatory cytokine IL-1β were significantly higher in LPS-challenged Ctr brains than in Hpa-tg brains (Fig. 2k). Heparanase overexpression thus attenuates recruitment of inflammatory cells into the brain and reduces activation of resident microglia in response to systemic LPS challenge.

### Delayed clearance of injected Aβ42 and poor recruitment of immune cells in Hpa-tg brain

**Fig. 3:**
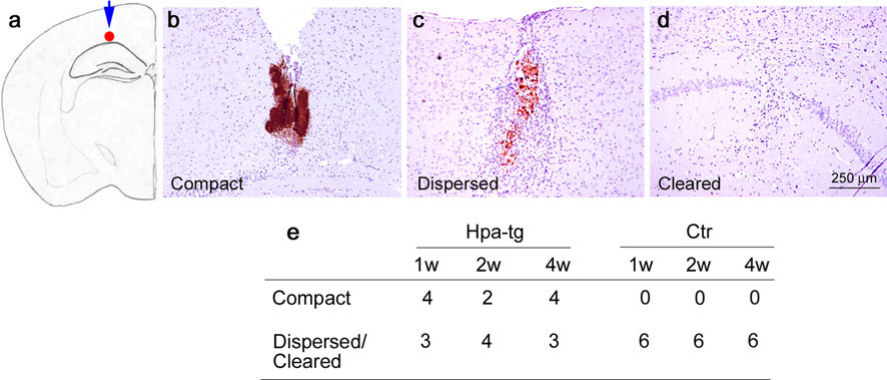
Aβ deposits following intracortical injection of aggregated synthetic human Aβ42 in Ctr and Hpa-tg mice. **a** Aβ42 injection site. **b** Representative compact deposit at the injection site from a Hpa-tg mouse. **c** Dispersed deposit, with scattered 6E10-positive aggregates. **d** Cleared deposit. **e** Number of Hpa-tg and Ctr brains presenting with each deposit state in groups killed after 1, 2, and 4 weeks

We also assessed the impact of heparanase overexpression on locally induced neuroinflammation elicited by intracortical injection of aggregated synthetic human Aβ42 (Fig. 3a). Groups of 6–7 Ctr and Hpa-tg mice were killed after 1, 2, and 4 weeks post-injection and brain sections were immunostained using the anti-Aβ antibody 6E10. The newly injected material appeared as compact, 6E10 (Fig. 3b) and Congo red (not shown) positive deposits that were dispersed (Fig. 3c) and ultimately cleared (Fig. 3d) over time. Clearance was markedly delayed in Hpa-tg compared to Ctr mice. Compact residual aggregates thus remained at the injection sites in 10 out of 20 Hpa-tg mice at time points when none were found in any of 18 Ctr mice (Fig. 3e).

**Fig. 4:**
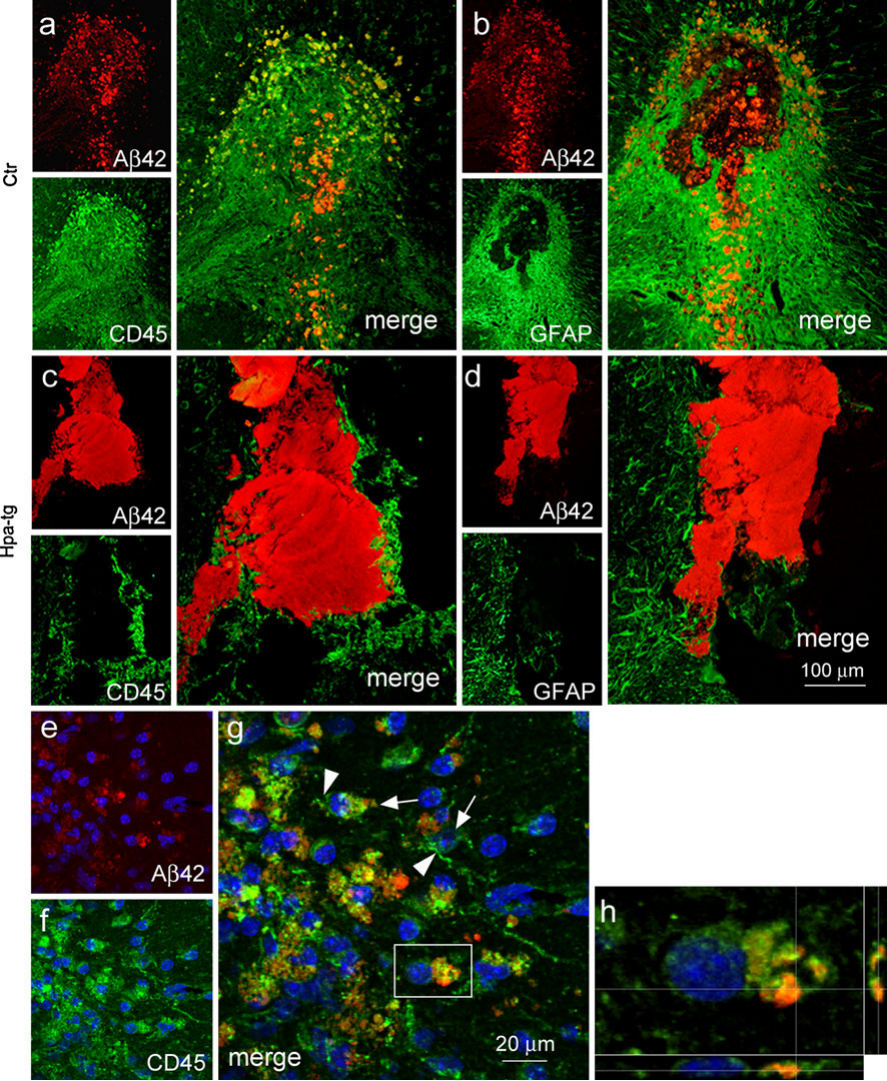
Cellular response to Aβ injection. Adjacent brain sections from mice, 2 weeks after Aβ42 injection were double immunostained with anti-Aβ42 for Aβ42 and anti-CD45 for macrophages or anti-GFAP for astrocytes. **a**, **b** Ctr mouse. Anti-Aβ42 stained dispersed Aβ deposits (**a**, **b** *red*), which were heavily infiltrated by CD45-positive macrophages (**a**, *green*), and surrounded by GFAP-positive astrocytes (**b** *green*). **c**, **d** Hpa-tg mouse. Anti-Aβ42 stained compact Aβ deposits (**c**, **d** *red*) with scant recruitment of CD45-positive macrophages (**c** *green*) and GFAP-positive astrocytes (**d** *green*). Confocal laser scanning microscopy of Aβ42 (**e**, *red*) and CD45 (**f**, *green*) immunostaining detects cells with amoeboid morphology (**g**, *arrow*) and few short processes (**g**, *arrowhead*). Aβ was engulfed by several CD45-positive cells. **h** Confocal microscopy of phagocytosed Aβ in a CD45-positive phagocyte indentified by the frame in **g**

To elucidate the reason for the impaired elimination of Aβ aggregates in the Hpa-tg brain, we examined injection sites for reactive cells using anti-CD45 antibody for detection of activated macrophages and anti-GFAP antibody for astrocytes. The dispersed Aβ deposits, found in Ctr mice, were heavily infiltrated by CD45-positive macrophages (Fig. 4a), and surrounded by GFAP-positive astrocytes (Fig. 4b), in contrast to the compact deposits in Hpa-tg mice that showed only scant recruitment of cells (Fig. 4c, d). Notably, dispersed deposits were also surrounded by fewer reactive cells in Hpa-tg than in Ctr mice (Supplementary Fig. 2). CD45-positive cells with amoeboid morphology were observed (4e-g), indicative of monocyte origin, similar to those detected after the LPS-induced inflammatory response. Confocal z-scan demonstrated internalization of the injected Aβ42 by CD45-positive macrophages, confirming their phagocytic phenotype (Fig. 4h). These data associate delayed degradation of the injected Aβ with an impaired inflammatory response.

**Fig. 5:**
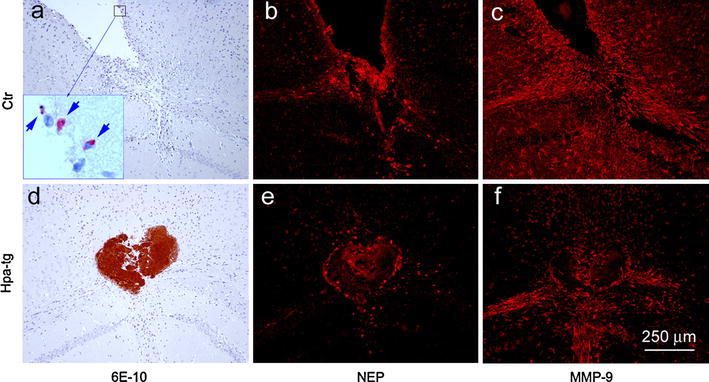
Expression of proteolytic enzymes following intracortical Aβ42 injection. Adjacent brain sections from a Ctr mouse and a Hpa-tg mouse 4 weeks after intracortical injection of Aβ42 were immunostained with the anti-Aβ antibody 6E10 (*red* in **a**, indicated by *arrows* in *insert,* and *red* in **d**), anti-NEP antibody (**b**, **e**) and anti-MMP-9 antibody (**c**, **f**), respectively. Immunosignals of NEP and MMP-9 were restricted to the injection sites, confined to smaller areas in Hpa-tg (**e**, **f**) than in Ctr brain (**b**, **c**)

Degradation of Aβ has been ascribed to proteases expressed by neurons, glial cells, macrophages and cerebral vasculature, in particular neprilysin (NEP) and matrix metalloproteinase-9 (MMP-9) [34]. To assess the impact of these enzymes on the degradation of injected Aβ, we examined the expression of NEP and MMP-9 in brain sections of a Ctr mouse that showed some remaining, dispersed Aβ (6E10-positive material, indicated by arrows in Fig. 5a; insert) and a Hpa-tg mouse retaining compact Aβ deposits (Fig. 5d). Immunosignals of NEP and MMP-9 were restricted to the injection sites, confined to smaller areas in Hpa-tg (Fig. 5e, f) than in Ctr brain (Fig. 5b, c). Double immunostaining of Ctr and Hpa-tg sections revealed expression of NEP mainly in CD45-positive macrophages, whereas MMP-9 appeared primarily associated with astrocytes (Supplementary Fig. 3). In accordance with these data, Ctr brain showed significantly higher NEP and MMP-9 protein levels than Hpa-tg brain, following systemic LPS challenge (Supplementary Fig. 1h, i). The lower expression of these enzymes in the Hpa-tg mouse brain thus correlates with a relative lack of activated macrophages and astrocytes. These data confirm that the delayed Aβ clearance in the heparanase-overexpressing brain is caused by impaired recruitment of inflammatory cells that degrade Aβ aggregates.

### Reduced expression of CCL2 and ICAM-1 in Hpa-tg brain

**Fig. 6:**
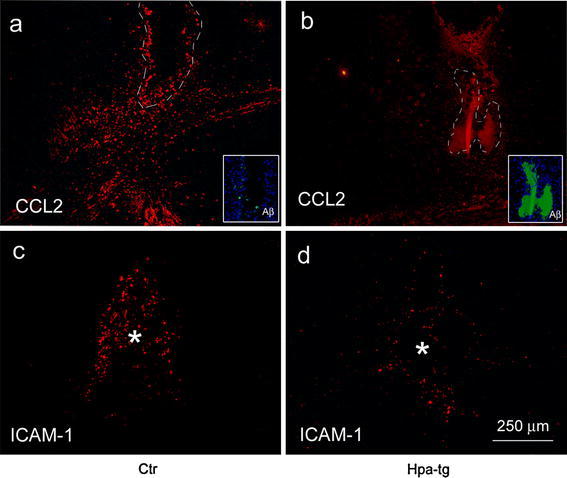
Expression of CCL2 and ICAM-1 at the intracortical Aβ42 injection site. **a**, **b** Brain sections from a Ctr mouse 4 weeks and a Hpa-tg mouse 2 weeks after Aβ42 injection were immunostained with anti-CCL2 antibody. The Ctr mouse showed pronounced cell-associated CCL2 immunosignals close to the injection site (**a** dispersed deposit) compared with scant signals in a Hpa-tg brain section (**b** compact deposit). The sites of the initial deposits are identified by *dashed lines*; the *inserts* show residual Aβ deposits (*green*) detected by the anti-Aβ antibody 6E10. The *red* signal surrounded by the *dashed line* in **b** is not associated with cells and is likely due to nonspecific fluorescence. **c**, **d** Brain tissue sections from a Ctr mouse as shown in Fig. 3c and a Hpa-tg mouse as shown in Fig. 5d were immunostained with anti-ICAM-1 antibody. ICAM-1 immunosignals restricted to the injection site (indicated by *asterisk*) were pronounced in Ctr brain (**c**) but scarce in Hpa-tg brain (**d**)

The above results demonstrated impaired infiltration and activation of immune cells in the Hpa-tg brain. Extravasation of leukocytes from blood to affected tissues in inflammation depends on leukocyte/endothelial cell recognition, which involves soluble chemotactic factors as well as receptors and ligands on cell surfaces [4]. Expression of the chemotactic protein CCL2 was examined in brain sections of a Ctr mouse with some residual, dispersed Aβ (Fig. 6a; insert) and a Hpa-tg mouse retaining compact Aβ deposits (Fig. 6b; insert). CCL2 immunosignals were restricted to the injection site and were much more pronounced in Ctr than in Hpa-tg brain (Fig. 6a, b). CCL2 appeared predominantly associated with CD45-positive macrophages in both Ctr and Hpa-tg animals (Supplementary Fig. 4).

**Fig. 7:**
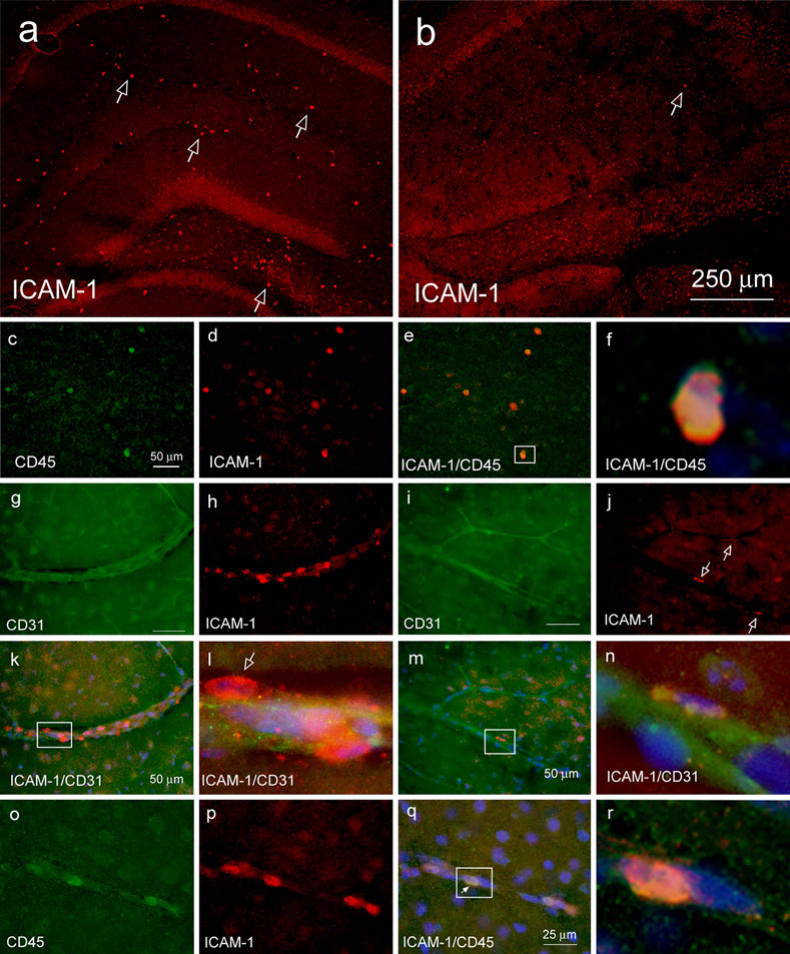
Expression of ICAM-1 by CD45-positive macrophages in brains of mice 20 h after intraperitoneal LPS challenge. **a**–**f** Parenchymal CD45-positive macrophages expressing ICAM-1. Brain tissue sections of a Ctr and a Hpa-tg mouse challenged by LPS were immunostained with anti-ICAM-1 (**a**–**b**) or double immunostained with anti-ICAM-1/CD45 antibodies (**c**–**f**). **a** Abundant cell-associated ICAM-1 immunosignals in the hippocampus of the Ctr mouse (some of the cells are indicated by *arrows*), **b** scant ICAM-1-positive cells in the hippocampus of the Hpa-tg mouse. Note: Similar nonspecific background fluorescence from the CA regions and dentate gyrus. **c** amoeboid CD45-positive macrophages, **d** ICAM-1 immunosignals, **e** overlay of ICAM-1 immunosignals with CD45-positive macrophages. **f** Enlarged image of a CD45-positive macrophage expressing ICAM-1 as framed in **e**. **g**–**n** Expression of ICAM-1 in parenchymal microvasculature. Brain tissue sections of the same animals as above were double immunostained with anti-CD31/anti-ICAM-1 antibodies. **g**, **i** Microvasculature stained by CD31 in Ctr and Hpa-tg mouse, respectively. **h** ICAM-1 immunosignals in Ctr mouse microvasculature. **j** ICAM-1 immunosignals in Hpa-tg mouse microvasculature (*arrows* point ICAM-1 immunosignals). **k** Abundant ICAM-1-positive cells associated with a micro-blood vessel in Ctr brain. **m** In contrast, a Hpa-tg micro-blood vessel is almost devoid of ICAM-1-positive cells. **l**, **n** Enlarged image of the frame in **k** and **m**, respectively. An *arrow* in **l** points to an ICAM-1-positive cell associated with the parenchymal abluminal surface of the blood vessel. **o**–**r** Expression of ICAM-1 by blood-borne macrophages associated with microvasculature. **o** CD45-positive blood-borne monocytes/macrophages. **p** ICAM-1 immunosignals. **q** Overlay of ICAM-1 immunosignals with CD45-positive monocyte/macrophages. *Arrow* indicates expression of ICAM-1 by a CD45-positive cell still within the blood vessel. **r** Enlarged image of a CD45 positive macrophage expressing ICAM-1 as framed in **q**

The transmembrane protein ICAM-1 (intercellular adhesion molecule 1, also known as CD54) is constitutively expressed at low levels on the cell surface of various cells types including endothelial cells and neutrophils, but dramatically upregulated in response to cytokines and LPS [8, 29, 30, 36, 47, 61]. We assessed ICAM-1 expression in brain tissue sections of LPS-challenged mice by immunostaining with anti-ICAM-1 antibody. Ctr brain, with upregulated IL-1β generation (Fig. 2k), showed ICAM-1-positive cells in the parenchyma (Fig. 7a). By contrast, Hpa-tg brain, with attenuated IL-1β response to LPS (Fig. 2k), was essentially devoid of ICAM-1-positive cells in the parenchyma (Fig. 7b). Double immunostaining with anti-CD31 antibody for blood vessel and ICAM-1 antibody showed ICAM-positive cells associated with microvasculature in Ctr parenchyma (Fig. 7g–h, k–l), but few such cells were found associated with Hpa-tg microvasculature (Fig. 7i–j, m–n). Double immunostaining with anti-CD45 and anti-ICAM-1 antibodies revealed that the ICAM-1-positive cells in the parenchyma (Fig. 7c–f) and the ICAM-1 positive cells associated with blood vessel walls (Fig. 7o–r) were also CD45-positive; hence the parenchymal cells are likely derived from blood-borne monocytes. These data are consistent with the view that ICAM-1 expression remains low until monocytes are in the process of leaving the circulation [37]. We did not detect clear upregulation of ICAM-1 on endothelial cells. It has been reported that ICAM-1 expression on monocytes mediates trans-endothelial migration [54]; thus the inhibited expression of ICAM-1 may be attributed to the impaired recruitment of CD45-positive macrophages in Hpa-tg mouse. CD45-associated ICAM-1 expression was also observed in brain sections of mice that had received intracortical Aβ42 injection (Supplementary Fig. 5) with more pronounced ICAM-1 immunosignals around the injection site in Ctr mice (Fig. 6c) than in Hpa-tg mice (Fig. 6d).

### Impaired transmigration of blood-borne monocytes across an in vitro model of the Hpa-tg blood–brain barrier

**Fig. 8:**
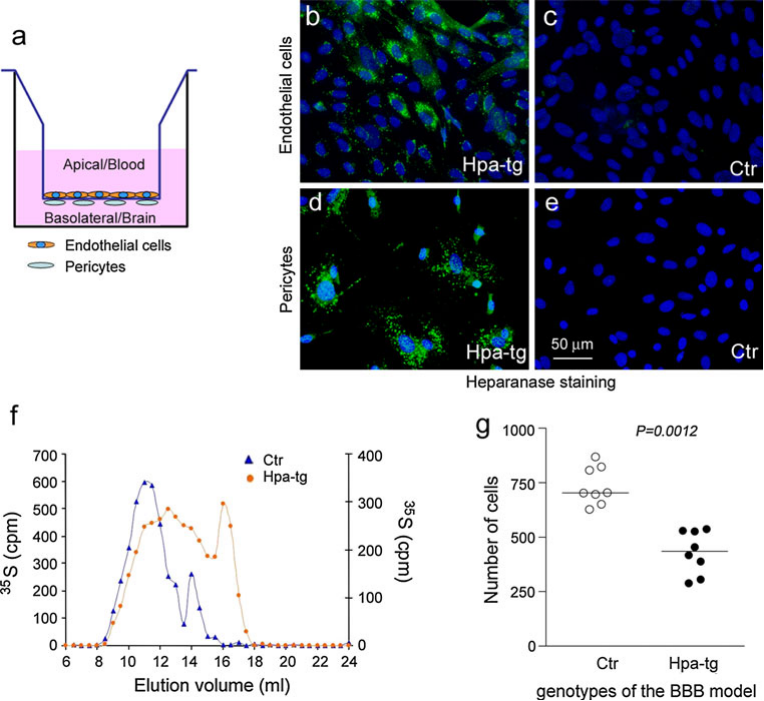
Monocyte transmigration assay using an in vitro BBB model. **a** Diagram of the BBB model assembled in cell-culture transwell inserts with primary endothelial cells (apical site) and pericytes (basolateral site) isolated from mouse brains. Heparanase overexpression in Hpa-tg endothelial cells (**b**) and pericytes (**d**) compared with Ctr cells (**c**, **e**) was verified by immunostaining with anti-heparanase antibody 733 (*green*). **f** Gel chromatography (Superose 12) showed a pronounced reduction in size of metabolically ^35^S-labeled HS free chains purified from Hpa-tg primary endothelial cells compared with HS from Ctr endothelial cells. **g** Number of monocytes transmigrated from the apical to the basolateral compartment containing CCL2 (50 ng/ml)

Endothelial HS is essential for leukocyte trafficking, as demonstrated by impaired neutrophil infiltration in various inflammation models due to deficient HS sulfation [60]. We therefore examined whether the scant recruitment of CD45-positive cells into the brain of Aβ-injected Hpa-tg mice could be caused by derangement of HS chains on cerebral microvascular endothelial cells. To this end, we prepared primary cerebral endothelial cells and pericytes from Hpa-tg and Ctr mice (Supplementary Fig. 6a-c) and assembled an in vitro BBB model to study monocyte transmigration (Fig. 8a). Both endothelial cells and pericytes from Hpa-tg mice showed distinct overexpression of heparanase (Fig. 8b–e). Similar to the HS isolated from whole brain (Fig. 1c), the Hpa-tg endothelial HS was truncated compared to Ctr HS chains (Fig. 8f). Monocytes, isolated from Ctr mice expressing green fluorescent protein (GFP) were added to the apical compartment of the transmigration device, and their migration across the cellular barrier was determined in response to CCL2 added to the basolateral compartment (Fig. 8a). The number of GFP monocytes settling in the basolateral chamber was significantly reduced in the Hpa-tg compared to the Ctr BBB model (Fig. 8g; Supplementary Fig. 7). Control experiments performed without addition of CCL2 showed minimal numbers of monocytes crossing the insert (data not shown), proving the active role of the chemoattractant in the transmigration event. These data demonstrate that perturbation of endothelial HS by heparanase significantly impedes transmigration of blood-borne monocytes across the BBB.

## Discussion

Proliferation and recruitment of activated immune cells in the brain parenchyma, including macrophages derived from microglia or from blood-borne monocytes, is a hallmark of neuroinflammation. The inflammatory state may serve to eliminate pathogens, dead cells and tissue debris after various acute assaults, but are also conspicuous in several neurodegenerative diseases, including AD [15, 35, 46]. An inflammatory stimulus typically induces cytokines such as IL-1β or TNF-α, which in turn activates a variety of cells (endothelial cells, microglia/macrophages, astrocytes) to release cell-attractant chemokines such as CCL2 [6, 23], and up-regulate expression of cell adhesion molecules such as ICAM-1 [29].

Previous studies have implicated endothelial HS in various inflammation models [60]. HS may function as a ligand for leukocyte L-selectin, as a carrier in chemokine transcytosis, and as a scaffold for presenting chemokines at the luminal surface of the endothelium. In the present work, we have specifically studied the various roles of HS in neuroinflammation by employing Hpa-tg mice that overexpress heparanase in multiple regions of the CNS, thus having HSPGs with truncated HS side chains (Fig. 1). Two inflammation models were assessed, one involving systemic LPS challenge, the other a local microinjection of fibrillar Aβ42. Ctr mice subjected to intraperitoneal LPS injection showed increased levels of cerebral IL-1β (Fig. 2k) and an abundance of monocytes adhering to and migrating across the wall of cerebral microvasculature (Fig. 2a–g), where they differentiated into CD45/ICAM-1 positive macrophages (Fig. 7). Moreover, there was massive activation of microglia to macrophage-like state as shown by up-regulated CD45 expression and ramified morphology (Fig. 2h–j). All of these effects were significantly attenuated in Hpa-tg mice (shown in the corresponding figures).

The effects of injected fibrillar Aβ, as expected, were essentially confined to brain areas in close vicinity to the protein deposition. Ctr mice showed massive infiltration of activated immune cells, including macrophages (presumably derived from both resident microglia and blood-borne monocytes) and astrocytes (Fig. 4). These CD45-positive macrophages expressed CCL2 (Supplementary Fig. 4), a chemoattractant with a central role in the recruitment of blood-borne monocytes to sites of tissue injury. Expression of the proteolytic enzymes, NEP and MMP-9 (Fig. 5; Supplementary Fig. 3), together with phagocytosis of Aβ by macrophages (Fig. 4e–h) presumably contributed to efficient dispersion of the Aβ deposits following intracortical injection (Fig. 4). Again, heparanase expression strongly attenuated all of these responses to challenge.

**Fig. 9:**
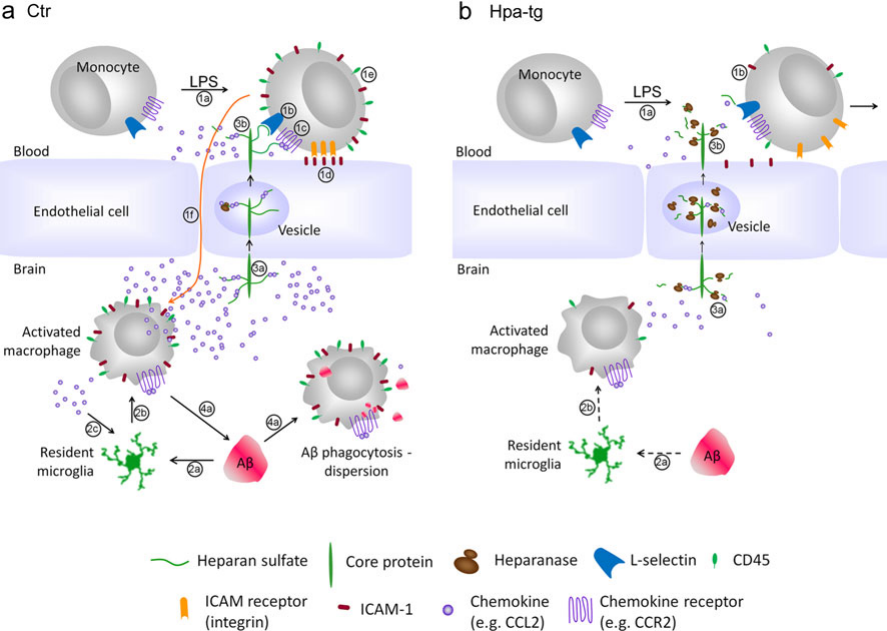
Schematic illustration of immune cell recruitment in response to LPS challenge and to microinjection of aggregated Aβ, illustrating the proposed inhibitory effect of heparanase. **a** Ctr: *1a*–*1f* transmigration of blood-borne monocytes across the endothelial barrier in response to systemic LPS challenge. *1a* LPS challenge; *1b* monocyte tethering through interactions of cell-surface L-selectin with HS for cell rolling; *1c* monocyte activation by HS-bound chemokine oligomers (e.g. CCL2) binding to specific receptors (CCR2); *1d* monocyte adhesion to endothelium through interaction of endothelial ICAM-1 with monocyte ICAM-1 receptors; *1e* monocytes upregulate CD45 and ICAM-1; *1f* monocytes transmigrate through the endothelial barrier into the brain, where they differentiate into activated macrophages and release cytokines (e.g. IL-1β) and chemokines (e.g. CCL2). *2a*–*2c* Activation of resident microglia in response to Aβ injection: *2a* Aβ deposit stimulates differentiation of resident microglia (*2b*) into activated macrophages; *2c* Cytokines released by the macrophages promote further activation and differentiation of resident microglia. Similar transition may be elicited by cytokines/chemokines released due to LPS stimulation. *3a*–*3b* Chemokines bind to HS chains of HSPGs at the abluminal surface of the endothelium, and are relayed by transcytosis to the luminal surface where they are presented to their receptors on tethered monocytes. *4a* Activated macrophages, presumably derived mainly from blood-borne monocytes, disperse the Aβ deposits through phagocytosis and proteolytic degradation. **b** Hpa-tg: Contrary to Ctr mice, Hpa-tg mice showed reduced expression of (*1b*) CD45 and ICAM-1 in response to LPS challenge (*1a*), as well as reduced response of resident microglia to Aβ injection (*dashed arrows* *2a*, *2b*). *3a*–*3b* Fragmentation of HS chains in the Hpa-tg brain downregulates binding of chemokines to HSPGs. These monocytes fail to attach and transmigrate, resulting in impaired Aβ clearance

A schematic overview of relevant steps of the neuroinflammatory process is provided in Fig. 9a, along with the consequences of heparanase overexpression in Fig. 9b. Such effects, presumably due to truncation of HS chains, are discerned at several stages. Interaction of endothelial HSPGs with L-selectin contributes to the initial stages of monocyte tethering to the vascular endothelium [60]. HS chains of sufficient length are required for presentation of chemokines to monocyte receptors to arrest the cells, and ensure firm adhesion of the cells to the endothelium through interaction of ICAM-1 on the endothelium with ICAM-1 receptors on monocytes, a key step in diapedesis. Moreover, HSPGs serve as carriers of chemokines in transcytosis from the parenchymal abluminal to the luminal aspect of the vascular endothelium [40]. Activation of resident microglia may be induced by pro-inflammatory molecules [21]), but also through direct interaction between Aβ and cell-surface HSPG [17]. Notably, LPS-induced upregulation of TNF-α and IL-1β was inhibited in Hpa-tg primary microglia compared with Ctr cells (our unpublished data). The poor response of Hpa-tg mice to either systemic LPS challenge or local Aβ injection, with scant recruitment of activated macrophages from blood-borne monocytes and resident microglia is thus due to failing HSPG support. Our data implicate heparanase as a potential key modulator of neuroinflammation.

The critical role for endothelial HS in trans-BBB diapedesis, suggested by the attenuating effects of heparanase, was supported by experiments involving an in vitro model of monocyte transmigration across a BBB using primary cerebral vascular cells. Cells derived from Hpa-tg mice overexpressed heparanase (Fig. 8b–e) and displayed truncated HS chains compared to Ctr cells (Fig. 8f). The passage of monocytes through the Hpa-tg BBB in response to CCL2 was significantly reduced relative to the Ctr BBB. Notably, chemokines, including CCL2, bind to HS as oligomers [26, 43] that occupy extended domains of the saccharide chain. For example, a dimer of the chemokine IL-8 requires a minimal sequence of 18 monosaccharide units for interaction [53]. The marked susceptibility of CCL2 function to HS cleavage is thus readily explained.

AD is a chronic disease, with Aβ deposition as a hallmark in the brain that is not simply modeled by microinjection of aggregated Aβ. Nevertheless, the effects of cerebral heparanase have potential bearing on various aspects of the disease. HS binds Aβ, supports fibril formation, and is typically part of Aβ deposits [39, 59]. Cell-surface HSPGs have been implicated in Aβ uptake and cytotoxicity [17, 28, 48]. These effects are, by and large, associated with disease pathogenesis, whereas our present findings expose HS also as potentially beneficial, contributing to recruitment and activation of immune cells that eliminate Aβ deposits. The HS/heparanase system is likely of key importance in the inflammatory response to AD. Endogenous heparanase is detected in the vasculature of the AD brain and of the Tg-2576 mouse model of Aβ deposition (Supplementary Fig. 8), thus it is present at the BBB and potentially capable of interfering with transmigration of phagocytes into the injured brain. In AD and the transgenic mouse models of AD, blood-derived macrophages associate with Aβ deposits [13, 31]. Such macrophages, recruited in response to cerebral injection of pre-aggregated Aβ, are thought to be better Aβ phagocytes than brain resident immune cells [10, 12, 24, 51], and poor infiltration of blood-borne macrophages may therefore contribute to Aβ accumulation [57]. Overexpressing heparanase in the brain of an AβPP-transgenic mouse by cross-breeding with a Hpa-tg mouse may clarify the relative roles of HS and heparanase in the Aβ pathology of AD.

## Electronic supplementary material

Below is the link to the electronic supplementary material.

Supplementary material 1 (TIFF 3795 kb)

Supplementary material 2 (TIFF 2295 kb)

Supplementary material 3 (TIFF 3107 kb)

Supplementary material 4 (TIFF 3923 kb)

Supplementary material 5 (TIFF 696 kb)

Supplementary material 6 (TIFF 1954 kb)

Supplementary material 7 (TIFF 339 kb)

Supplementary material 8 (TIFF 1811 kb)

Supplementary material 9 (DOC 45 kb)
