# Supplementary figures and images for: Heparanase overexpression impairs inflammatory response and macrophage-mediated clearance of amyloid-β in murine brain
Source: Acta Neuropathol. 2012 Jun 13;124(4):465–78. doi: 10.1007/s00401-012-0997-1 (PMC3444710; doi:10.1007/s00401-012-0997-1)

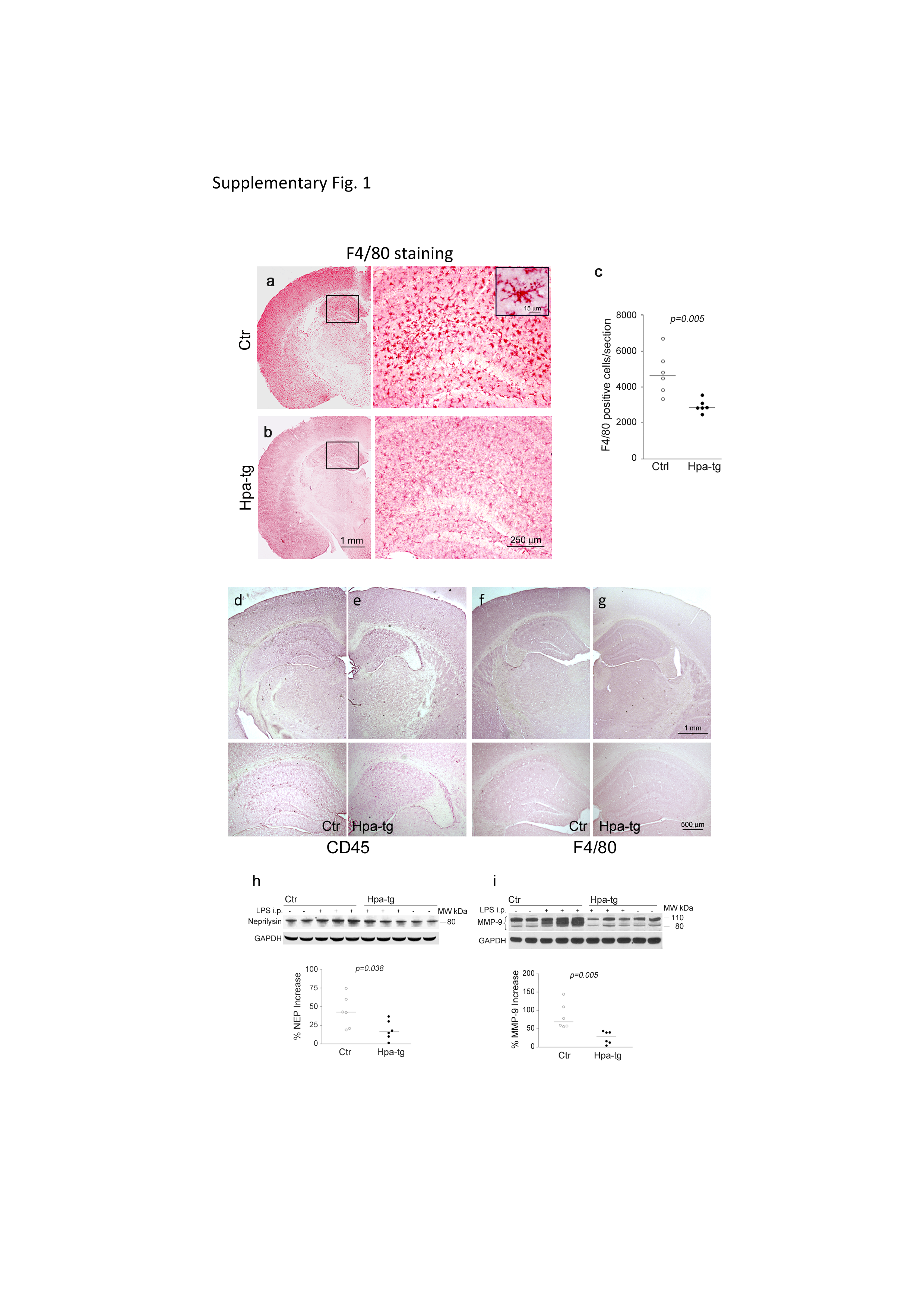

Supplement: Supplementary file 1 — Supplementary material 1 (TIFF 3795 kb) [file 401_2012_997_MOESM1_ESM.tif]

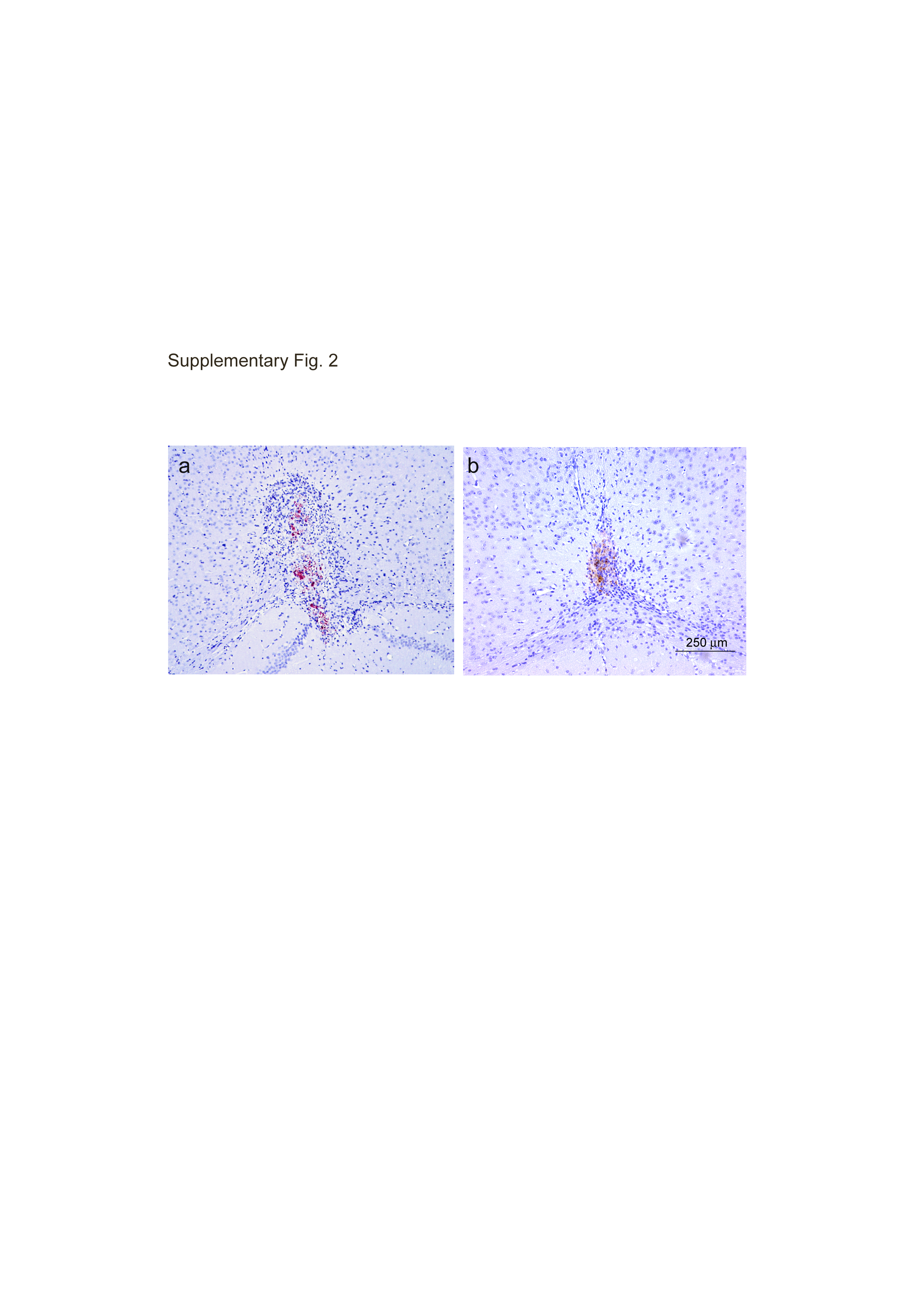

Supplement: Supplementary file 2 — Supplementary material 2 (TIFF 2295 kb) [file 401_2012_997_MOESM2_ESM.tif]

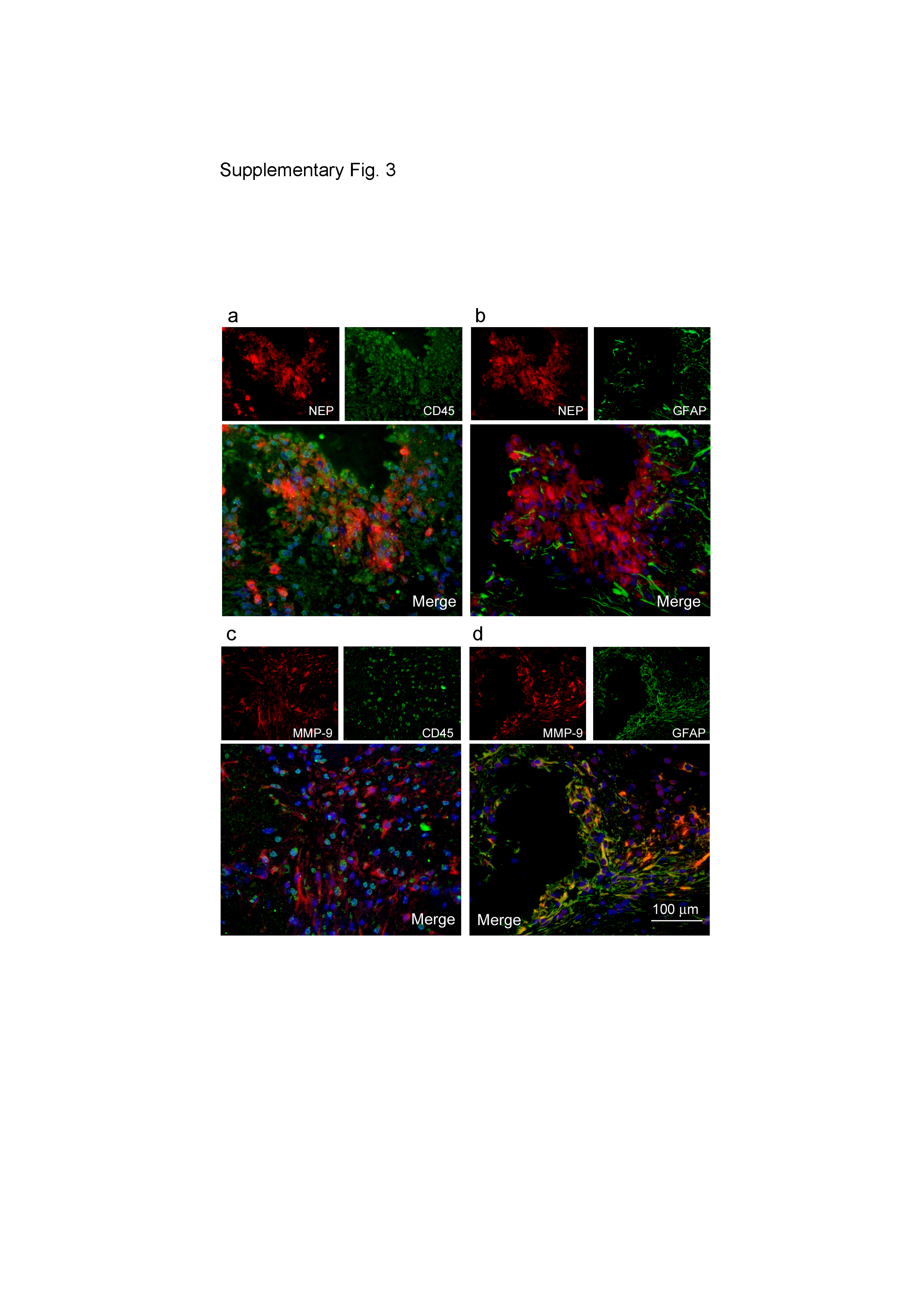

Supplement: Supplementary file 3 — Supplementary material 3 (TIFF 3107 kb) [file 401_2012_997_MOESM3_ESM.tif]

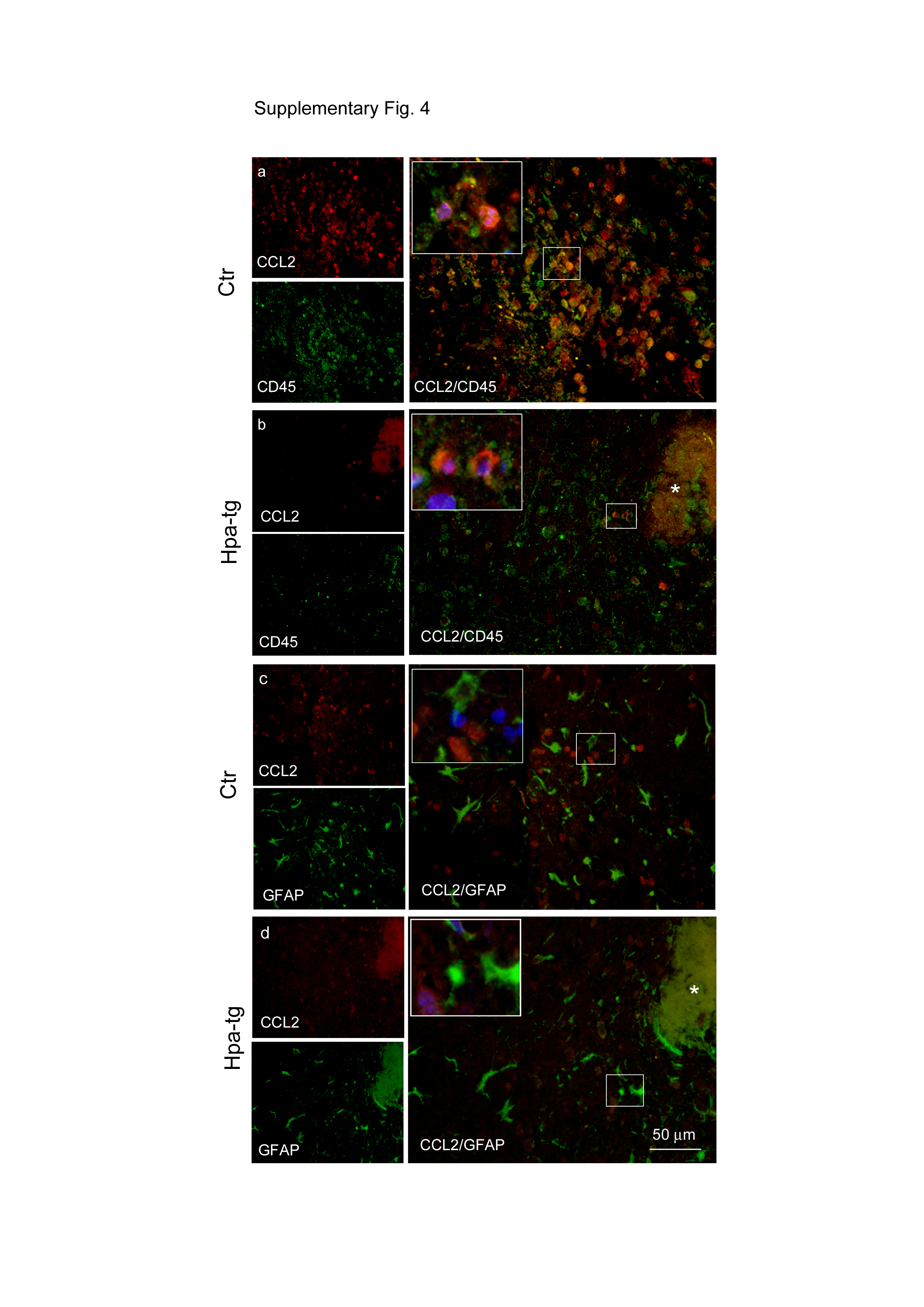

Supplement: Supplementary file 4 — Supplementary material 4 (TIFF 3923 kb) [file 401_2012_997_MOESM4_ESM.tif]

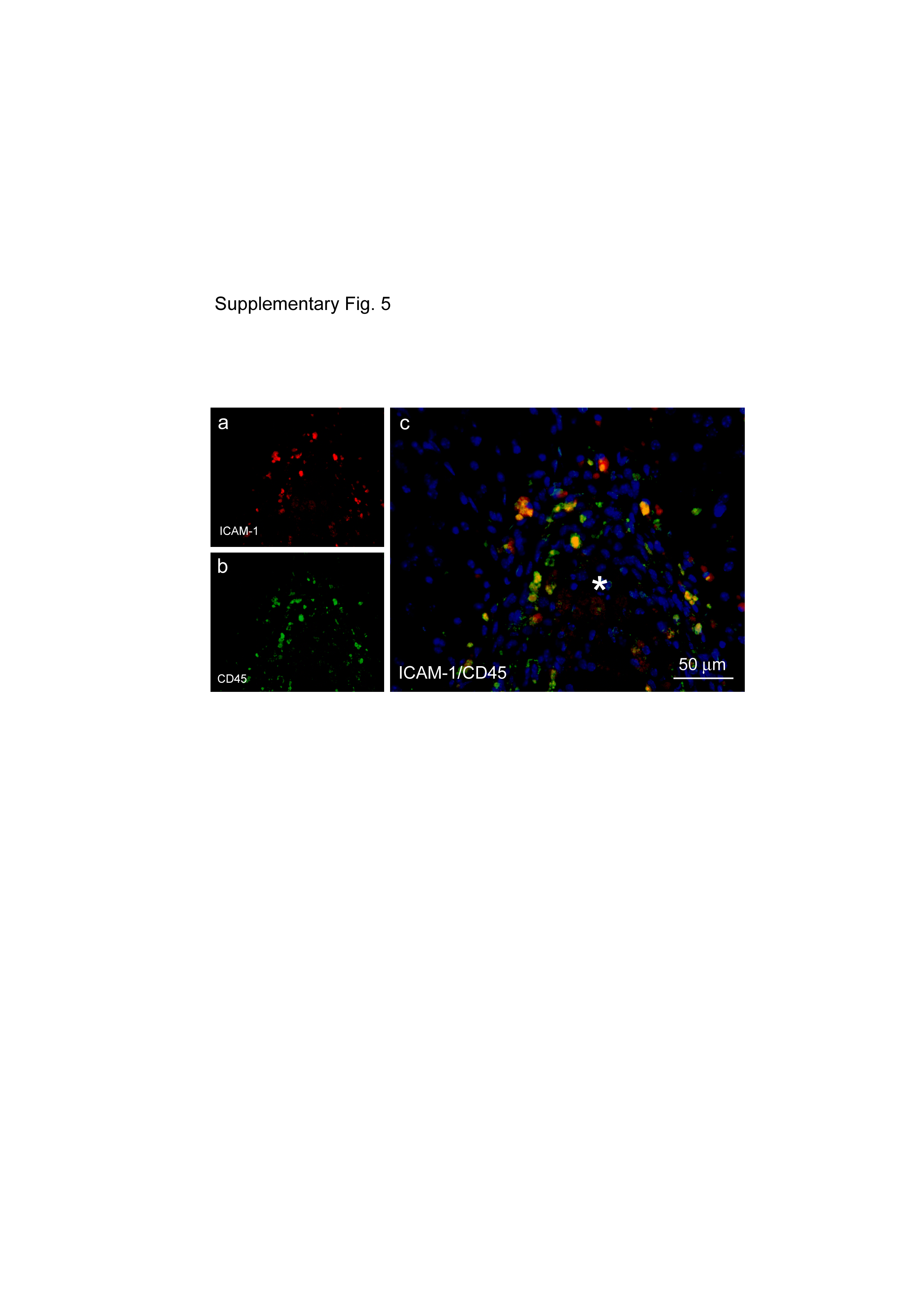

Supplement: Supplementary file 5 — Supplementary material 5 (TIFF 696 kb) [file 401_2012_997_MOESM5_ESM.tif]

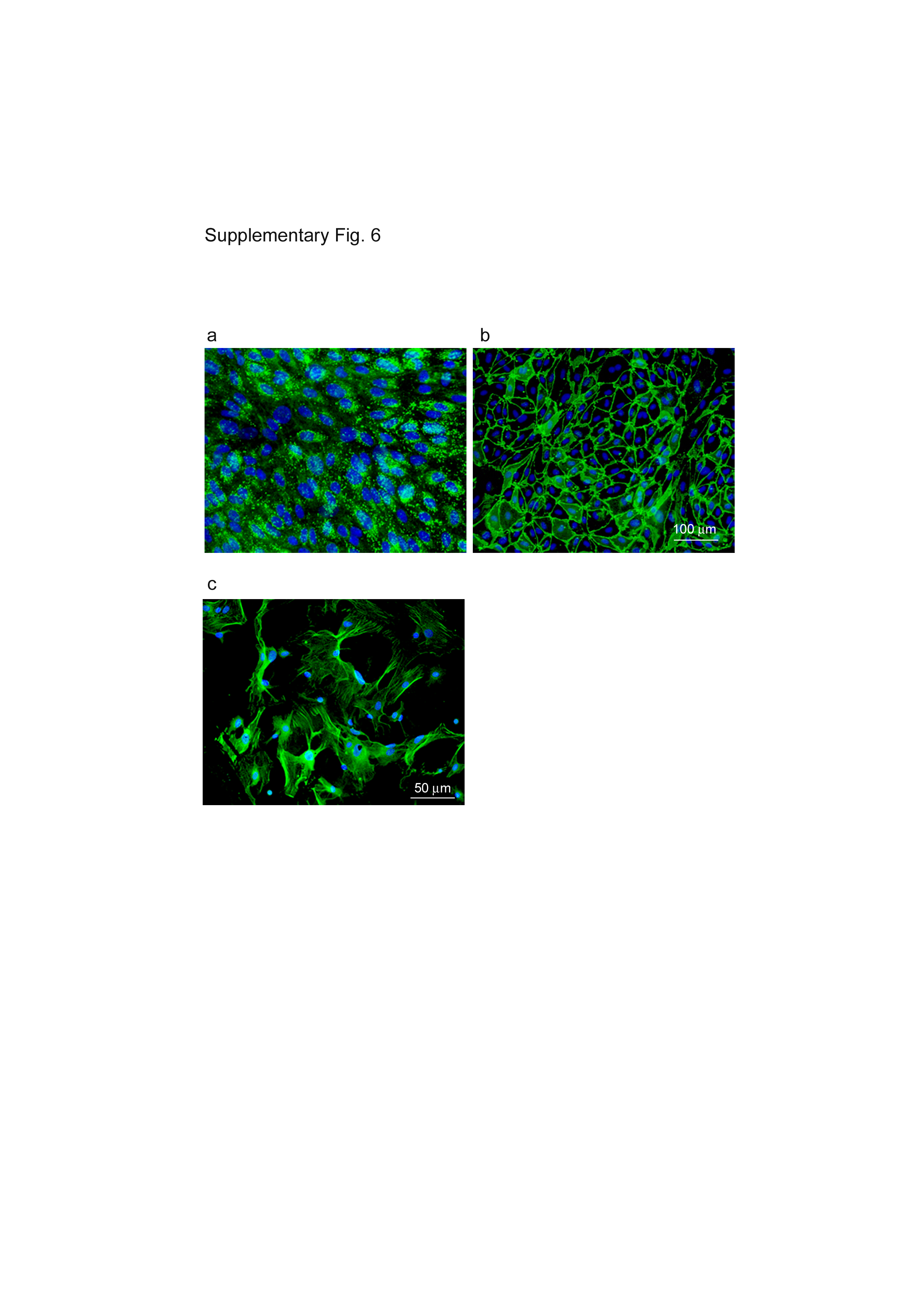

Supplement: Supplementary file 6 — Supplementary material 6 (TIFF 1954 kb) [file 401_2012_997_MOESM6_ESM.tif]

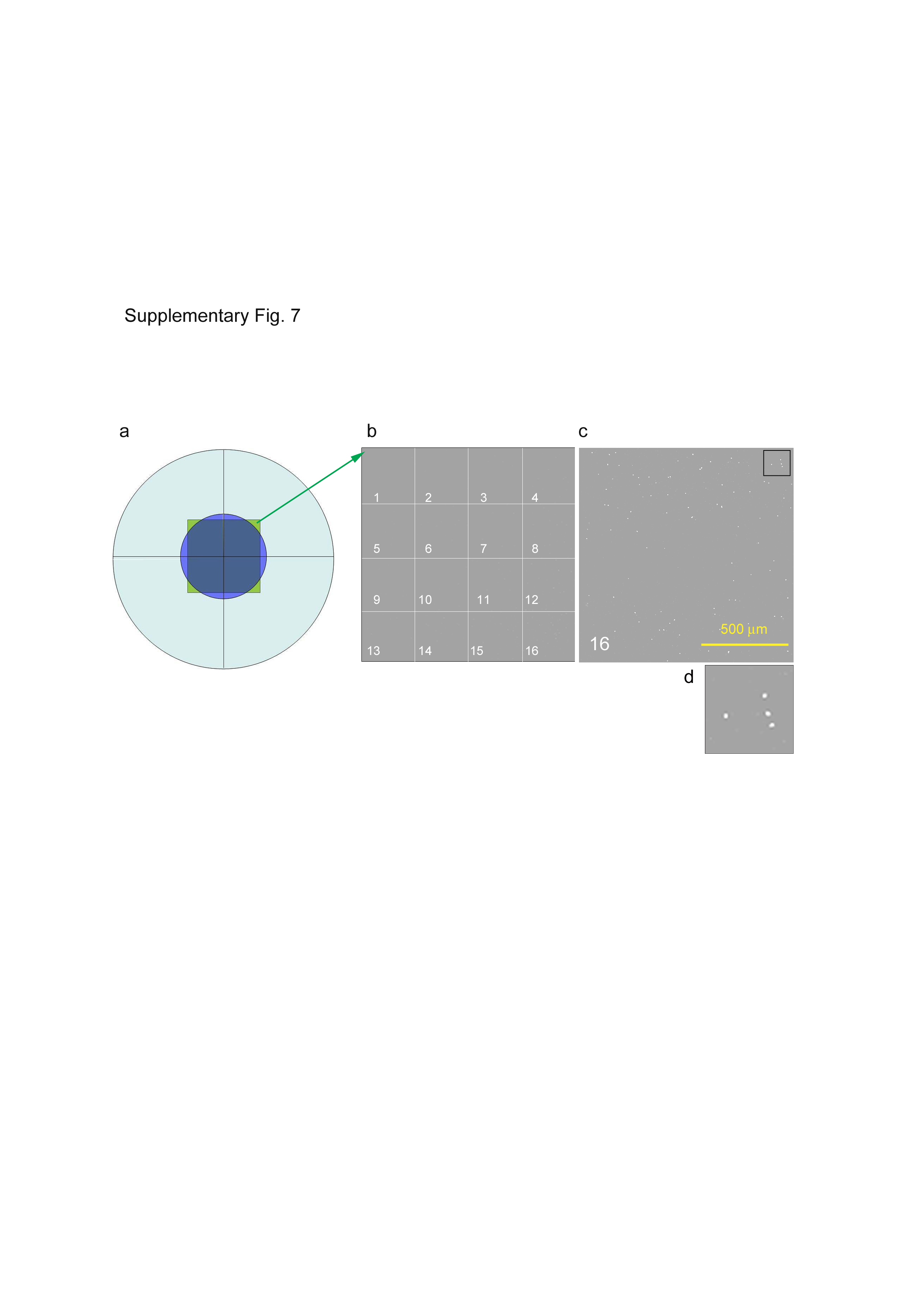

Supplement: Supplementary file 7 — Supplementary material 7 (TIFF 339 kb) [file 401_2012_997_MOESM7_ESM.tif]

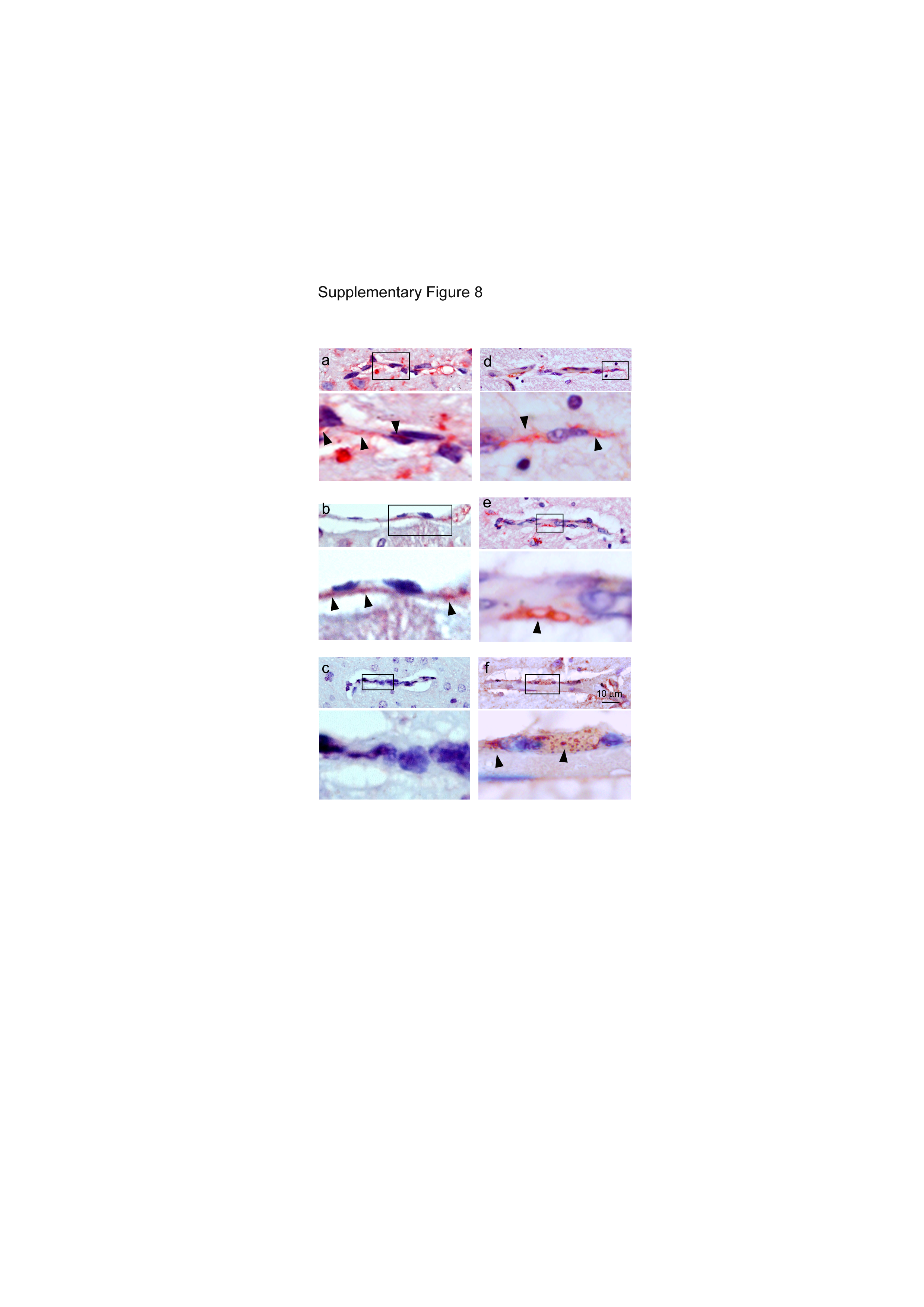

Supplement: Supplementary file 8 — Supplementary material 8 (TIFF 1811 kb) [file 401_2012_997_MOESM8_ESM.tif]
