# Supplementary material for: Heparanase overexpression impairs inflammatory response and macrophage-mediated clearance of amyloid-β in murine brain
Source: Acta Neuropathol. 2012 Jun 13;124(4):465–78. doi: 10.1007/s00401-012-0997-1 (PMC3444710; doi:10.1007/s00401-012-0997-1)
Supplement: Supplementary file 9 — Supplementary material 9 (DOC 45 kb) [file 401_2012_997_MOESM9_ESM.doc]

**Supplementary materials**

**Supplementary methods**

**Isolation and analysis of HSPGs**

For *in vivo* labeling, mice were injected intraperitoneally with 0.5 mCi Na_2_^35^SO_4_ (specific activity 1,500 Ci/mmol; Perkin Elmer, Waltham, MA) and maintained for 45 min with free access to water and food. Brains from sacrificed animals were dissected and homogenized with a Dounce homogenizer in 6 volumes of ice-cold 50 mM Tris-HCl, pH 7.4, 1% (v/v) Triton X-100, 4 M urea, 0.25 M NaCl and the supernatants were collected following centrifugation. For labeling endothelial cells, the primary cells isolated from the brain (see following) were cultured in the presence of 100 µCi Na_2_^35^SO_4_/ml for 24 hr. Then the medium was collected and the cells (total 1.5x10^6^) were lysed. For purification of HSPGs, the brain supernatant fractions, the cell culture medium fraction and the cell lysate were separately applied to DEAE-Sephacel columns equilibrated in 50 mM Tris-HCl, pH 7.4, 0.3 M NaCl. The columns were extensively washed with the same buffer and were then eluted with 1.5 M NaCl in the same buffer. The eluates were desalted, lyophilized and digested with chondroitinase ABC (Seikagaku, Tokyo, Japan) and benzonase (Merck, San Diego, CA). The digests were re-applied to DEAE-Sephacel columns to remove degraded chondroitin sulfate and oligonucleotides. The bound materials were eluted with 1.5 M NaCl and analyzed for molecular size by gel chromatography. For release of HS chains from the core protein, the samples were treated with 0.5 N NaOH at 4°C overnight, and were then neutralized and desalted. For disaccharide compositional analysis, samples were subjected to deaminative cleavage at *N*-sulfated glucosamine (GlcNS) residues by treatment with nitrous acid (pH 1.5) and reduced with NaB^3^H_4_. The resulting oligosaccharides were separated on a Superdex-30 column (GE Healthcare Biosciences) in 0.5 M NH_4_CO_3_. The disaccharide fraction was collected and analyzed on a Partisil-10 SAX column as previously described [1].

**Primary cell culture and *in vitro* BBB model**

Brain capillary endothelial cells and pericytes were prepared from 4-8 week-old Hpa-tg and Ctr mice, basically as described [3]. After removing meninges, the brain was chopped in ice-cold DMEM into small pieces that were incubated in DMEM containing 0.75mg/ml collagenase type 2 (Invitrogen), DNase (10u/ml, Sigma), gentamicin (50µg/ml) at 37°C with shaking for 10 min. The brain preparations were then dissociated thoroughly by pipetting through a 1 ml tip and further incubated for 50 min with shaking. After the incubation, the preparations were centrifuged at 1,500 x g for 10 min to obtain tissue pellets. To isolate capillaries, the pellets were centrifuged in DMEM containing 20% bovine serum albumin at 1,000 x g for 2 x 10 min at 4°C. The capillaries obtained were incubated in DMEM containing collagenase-dispase (1mg/ml, Roche) and DNAase (Sigma) (2.5µg/ml) for 60 min with shaking, and then endothelial cell clusters were isolated from the samples by centrifugation on a 40% continuous Percoll gradient (GE Healthcare). The cell clusters were washed with PBS and plated on cell culture plates coated with stabilized bovine fibronectin (Biomedical Technologies, Inc.). The cells were cultured for 2 days in DMEM/F12 medium containing 10% plasma derived serum (First Link UK limited), basic fibroblast growth factor (1.5ng/ml), heparin (100µg/ml), insulin-transferrin-selenium-X supplement 100x (10µl/ml), puromycin (4µg/ml, Invitrogen), gentamicin (5µg/ml, Invitrogen). From the 3^rd^ day, the cells were cultured in a medium essentially the same as above but without puromycin. On the 6^th^ day, endothelial cells were ready for constructing the *in vitro* BBB model. To obtain pericytes, the cell clusters were cultured in DMEM medium containing 10% FBS and gentamicin (5µg/ml) for 2 weeks.

**Monocyte preparation and transmigration assay**

Blood-borne monocytes were prepared from C57BL/6-Tg(UBC-GFP) mice that universally express the green fluorescent protein (GFP) (The JAX Lab). Mice were deeply anaesthetized (2.5% avertin 500µl/mouse i.p.) and blood (about 1ml/mouse) was collected into heparin-containing tubes. Monocytes were isolated from the blood by Ficoll Paque Plus (GE Healthcare Sweden) density gradient centrifugation. This preparation contains a large proportion of lymphocytes (>80%) and small proportion of monocytes (<20%). To preserve cell vitality no further purification was performed.

To the basal compartment (brain), 50 ng/ml of monocyte chemoattractant protein-1 (CCL2, also referred to as MCP-1) was added, then immediately after isolation the blood cells (120,000 cells/insert) were loaded into the apical compartment (blood). Monocytes migration from the apical compartment across the in vitro BBB into the basal compartment was monitored with a Zeiss confocal laser scanning microscope LSM 700/CO2 with a stage incubator (37°C, 5% CO_2_). Tile scan images (4 x 4) of 10 x magnification were taken. The image area is 0.262 cm^2^ (5,118.89 x 5,118.89 µm). The number of GFP-monocytes was counted with ImageJ software (Supporting Fig. S5). Control assay with no-cell inserts or omitting CCL2 were also performed.

The integrity of the BBB model was tested using Evans blue dye (EB) conjugated with BSA as described [2]. No leaking was observed (data not shown).

**Brain tissues**

Human brain tissues were obtained from the Netherlands Brain Bank and the Tg2576 mouse brains from AstraZeneca R&D, Södertälje, Sweden. The experimental protocols for the brain tissues were conducted in compliance with Swedish legislation.

**References**

1. Escobar Galvis ML, Jia J, Zhang X, Jastrebova N, Spillmann D, Gottfridsson E, van Kuppevelt TH, Zcharia E, Vlodavsky I, Lindahl U, Li JP (2007) Transgenic or tumor-induced expression of heparanase upregulates sulfation of heparan sulfate. Nat Chem Biol 3 (12):773-778

2. Moitra J, Sammani S, Garcia JG (2007) Re-evaluation of Evans Blue dye as a marker of albumin clearance in murine models of acute lung injury. Transl Res 150 (4):253-265

3. Nakagawa S, Deli MA, Kawaguchi H, Shimizudani T, Shimono T, Kittel A, Tanaka K, Niwa M (2009) A new blood-brain barrier model using primary rat brain endothelial cells, pericytes and astrocytes. Neurochem Int 54 (3-4):253-263

**Supplementary** figure legends

Supplementary Fig. 1 Impaired immune response of the Hpa-tg brain to LPS challenge. Four-month-old Hpa-tg and Ctr mice received LPS (5mg/kg) by intraperitoneal injection. The mice were sacrificed 20 hr after the treatment. **a, b** Brain tissue sections were immunostained with anti-F4/80 antibody for activated macrophages. Panels to the right are high-magnification images of the areas indicated by the frames in the left panels. Insert in the right panel of **a** shows a typical F4/80 positive macrophage in Ctr brain. **c** F4/80-positive macrophages were significantly fewer in Hpa-tg whole-brain sections than in Ctr sections. **d**-**g** Few inflammatory cells in brains of mice not challenged with LPS. **d, e** Brain sections of 4-month-old Ctr (**d)** and Hpa-tg (**e**) mice were immunostained with anti-CD45 antibody; no recruitment of CD-45 positive cells was observed. **f, g** Anti-F4/80 antibody immunostaining also failed to reveal any recruitment of ramified F4/80 macrophges in either Ctr (**f**) or Hpa-tg (**g**) brains. **h, i** Western blotting analyses of neprilysin (NEP) and matrix metallopeptidase 9 (MMP-9) in brain extracts. Upper panels: representative immunoblots; lower panels: relative increase of protein levels in LPS-challenged compared with non-treated animals, determined by densitometry. Protein levels of NEP and MMP-9 were significantly lower in Hpa-tg than in Ctr brains.

Supplementary Fig. 2 Reduced accumulation of inflammatory cells around dispersed Aβ deposits in Hpa-tg (b) compared to Ctr (a) brain. Sections were immunostained with anti-Aβ 6E10 antibody (red) and counterstained with haematoxylin (blue).

Supplementary Fig. 3 Expression of NEP by CD45-positive macrophages and MMP-9 by GFAP positive astrocytes following intracortical Aβ42 injection. Adjacent brain tissue sections as in Fig. 5 were double immunostained with anti-NEP/anti-CD45, anti-NEP/anti-GFAP, anti-MMP9/anti-CD45, and anti-MMP-9/anti-GFAP antibodies, respectively. a Association of NEP immunosignals with CD45-positive macrophages in Ctr brain. b No association of NEP immunosignals with GFAP-positive astrocytes in Ctr brain. c No association of MMP-9 immunosignals with CD45-positive macrophages in Hpa-tg brain. d Association of MMP-9 immunosignals with GFAP-positive astrocytes in Hpa-tg brain.

Supplementary Fig. 4 Expression of CCL2 by CD45-positive macrophages in the brains of mice following intracortical Aβ42 injection. Adjacent brain tissue sections of the animals as in Fig. 6a, b were double immunostained with anti-CCL2/anti-CD45 and anti-CCL2/anti-GFAP antibodies, respectively. Heavier infiltrations of CD45-positive macrophages and GFAP-positive astrocytes were seen in Ctr brain (a, c) compared to Hpa-tg brain (b, d). Immunosignals of CCL2 were restricted to the injection sites in both Ctr and Hpa-tg brains where CD45-positive cells were detected (**a, b**: right panels). CD45-positive macrophages appeared to be the only source of CCL2 in both Ctr and Hpa-tg brain sections (a, b), as no clear co-localization of GFAP and CCL2 immunosignals were observed (c, d). Inserts in the left upper corners are the enlarged images of the small frames in the corresponding figures. The asterisk in b or d indicates compact Aβ deposits in Hpa-tg mouse.

Supplementary Fig. 5 Association of ICAM-1 immunosignals with CD45-positive materials in the brain of a Ctr mouse two weeks of intracortical Aβ injection. a ICAM-1 immunosignals. b CD45 immunosignals. c Overlay of CD45 and ICAM-1 immunosignals. Asterisk indicates the injection site.

Supplementary Fig. 6 Primary endothelial cells and pericytes used to prepare the *in vitro* BBB model. a Anti-vWF antibody staining of Ctr endothelial cells. b Anti-claudin 5 staining of tight-junction protein claudin 5 produced by Ctr endothelial cells. c Anti-αSMA staining of Ctr pericytes. Immunostaining of the corresponding primary cells isolated from Hpa-tg brain gave similar results.

Supplementary Fig. 7 Analysis of monocyte transmigration. Monocytes settled on the bottom of the cell culture well after migration through the *in vitro* BBB model were monitored by scanning with a Zeiss laser-scanning microscope LSM 700/CO2 Microscope with a stage incubator (37°C, 5% CO2). a Diagram of the settings. Outer gray circle: the bottom area of a well in a 24-well culture plate; Blue circle: cell growth area of a Falcon™ HTS FluoroBlok™ insert; Green square: 4x4 tile scanning area (5,118.89 x 5,118.89 µm) resulting in 16 images of equal size (b); c High magnification of image 16; d Enlarged examples of 4 individual monocytes as framed insert in c

Supplementary Fig. 8 Vascular expression of heparanase in murine and human brains. Tissue sections were immunostained with anti-heparanase antibody 733. Parenchymal microvessels from a Hpa-tg mouse (a) and a Tg-2576 AβPP transgenic mouse (b) displayed 733 immunosignals (arrowheads, red). c No clear 733 immunosignals were discerned in parenchymal microvasculature from a Ctr mouse. d-e Similar heparanase staining of parenchymal microvessels was detected in an AD brain and f of an age-matched cognitively healthy brain (arrowheads, red), presumably duo to aging. Lower panels show enlargements of framed areas in corresponding upper panels.

**Supplementary Table 1: Primary antibodies used in the study**

**Antibody Host Type Specificity Concentration** **Source**

| Anti-heparanase (733) | Rabbit | poly | Human, also mouse  50 kDa active enzyme | IHC: 1:250 | Vlodavsky lab |
| --- | --- | --- | --- | --- | --- |
| 6E10 | Mouse | mono | Human and rodent Aβ & AβPP | IHC:0.25µg/ml | Signet Lab. USA |
| Anti-Aβ42 | Rabbit | poly | Human Aβ42  (C-terminal epitope specific) | IHC: 0.5µg/ml | BioSource, Belgium |
| Anti-Aβ40 | Rabbit | poly | Human Aβ40  (C-terminal epitope specific) | IHC: 0.5µg/ml | BioSource, Belgium |
| Anti-F4/80 | Rat | mono | Mouse macrophage F4/80 antigen | IHC: 2.5µg/ml | AbD Serotec, UK |
| Anti-CD45 | Rat | mono | CD45 | IHC: 5µg/ml | R&D systems, UK |
| Anti-GFAP | Mouse | mono | Glial fibrillary acidic protein | IHC: 0.1µg/ml | Sigma, USA |
| Anti-IL-1β | Rabbit | poly | Mouse IL-1β | WB: 0.2µg/ml | abcam, UK |
| Anti-MMP-9 | Rabbit | poly | Mouse and human MMP-9 | WB: 0.4µg/ml | abcam, UK |
| Anti-NEP | Rat | mono | Mouse and human neprilysin | IHC: 5µg/ml | R&D systems, UK |
| Anti-vWF | Rabbit | poly | Mouse von Willebrand Factor | ICC: 0.5 µg/ml | Chemicon, USA |
| Anti-Claudin 5 | Rabbit | poly | Human, mouse, rat Claudin 5 protein | ICC: 1 µg/ml | abcam, UK |
| Anti-αSMA | Rabbit | poly | Human, mouse, rat  α smooth muscle actin | ICC: 1:100 | abcam, UK |
| Anti-CD31 | Rat | mono | Mouse CD31 | IHC: 2µg/ml | BD Pharmingen  Belgium |
| Anti-ICAM-1 (CD54) | Rabbit | poly | Mouse, rat ICAM-1 | IHC: 5µg/ml | ABBIOTEC, USA |
| Anit-CCL2 | Rabbit | poly | Mouse CCL2 | IHC: 2µg/ml | Santa Cruz, USA |
| Anti-GAPDH | Mouse | mono | Human and rodent  glyceraldehyde-3- phosphate dehydrogenase | WB: 0.1µg/ml | Santa Cruz, Germany |

IHC: Immunohistochemistry; ICC: Immunocytochemistry; WB: Western blotting
